# The Impact of Shift Work on Sleep, Alertness and Performance in Healthcare Workers

**DOI:** 10.1038/s41598-019-40914-x

**Published:** 2019-03-15

**Authors:** Saranea Ganesan, Michelle Magee, Julia E. Stone, Megan D. Mulhall, Allison Collins, Mark E. Howard, Steven W. Lockley, Shantha M. W. Rajaratnam, Tracey L. Sletten

**Affiliations:** 1 Cooperative Research Centre for Alertness, Safety and Productivity, Melbourne, Victoria Australia; 2 Monash Institute of Cognitive and Clinical Neurosciences and School of Psychological Sciences, Monash University, Clayton, Victoria Australia; 3 Institute for Breathing and Sleep, Austin Health, Heidelberg, Victoria Australia; 4 Division of Sleep and Circadian Disorders, Department of Medicine, Brigham and Women’s Hospital, Boston, MA USA; 5 Division of Sleep Medicine, Harvard Medical School, Boston, MA USA

## Abstract

Shift work is associated with impaired alertness and performance due to sleep loss and circadian misalignment. This study examined sleep between shift types (day, evening, night), and alertness and performance during day and night shifts in 52 intensive care workers. Sleep and wake duration between shifts were evaluated using wrist actigraphs and diaries. Subjective sleepiness (Karolinska Sleepiness Scale, KSS) and Psychomotor Vigilance Test (PVT) performance were examined during day shift, and on the first and subsequent night shifts (3^rd^, 4^th^ or 5^th^). Circadian phase was assessed using urinary 6-sulphatoxymelatonin rhythms. Sleep was most restricted between consecutive night shifts (5.74 ± 1.30 h), consecutive day shifts (5.83 ± 0.92 h) and between evening and day shifts (5.20 ± 0.90 h). KSS and PVT mean reaction times were higher at the end of the first and subsequent night shift compared to day shift, with KSS highest at the end of the first night. On nights, working during the circadian acrophase of the urinary melatonin rhythm led to poorer outcomes on the KSS and PVT. In rotating shift workers, early day shifts can be associated with similar sleep restriction to night shifts, particularly when scheduled immediately following an evening shift. Alertness and performance remain most impaired during night shifts given the lack of circadian adaptation to night work. Although healthcare workers perceive themselves to be less alert on the first night shift compared to subsequent night shifts, objective performance is equally impaired on subsequent nights.

## Introduction

In many countries, healthcare workers make up the single largest proportion of shift workers^1–3^. To facilitate the provision of 24 hour emergency healthcare services and hospital care for the critically unwell, shift work comprising irregular work hours outside of traditional diurnal work times is widely adopted^4^. These work hours pose a challenge to the healthcare industry as shift work is likely to have major negative implications on patient care and patient safety^5–7^, in addition to its association with significant economic and productivity costs^8,9^.

Misalignment of the circadian pacemaker with sleep-wake timing is common in shift workers, particularly during nights^10^ and results in sleep loss^11,12^ and excessive sleepiness during work shifts^13^. The night shift is often associated with extended episodes of wakefulness^14–16^, particularly on the first night in a series when an individual may wake at a normal time in the morning, and remain awake during the day prior to starting the first night^15^. Other shift work schedules, which may involve early start or late end times, may also impact sleep duration and increase sleep-wake disturbances^17,18^. The combined effect of these circadian and sleep-related factors impair alertness and performance while on duty^16,19^, and often impact on safe driving practices during the commute to and from work^20–22^.

The time available for recovery sleep prior to and between shifts is an important factor influencing performance during subsequent shifts. Shift workers in the healthcare industry rotate between different shift times^23^, potentially limiting recovery sleep, particularly when transitioning from shifts that end late and start early the next morning^18,24^. In recognition of this risk, the European Working Time Directive introduced the requirement for at least 11 hours of rest between shifts^25^ including in healthcare^26,27^ to allow sufficient time to commute to and from work and still provide adequate time for sleep. For nurses and doctors in Australia, regulatory guidelines which provide for sufficient rest between shifts, and a limit on the number of consecutive hours worked during a shift is yet to be implemented. As a consequence, Australian healthcare workers engage in a wide variety of hours and under a broad range of roster structures. Therefore, the extent to which these factors interact to affect sleep, alertness and performance has important health and safety implications.

The timing of the sleep opportunity and work period are vital in determining the quantity and quality of sleep and performance at work^28^. The circadian system promotes sleep at night and wake during the day, resulting in a shorter sleep duration between night shifts^29^ and furthermore, cumulative sleep loss over successive night shifts. There is generally little circadian adaptation to negate these effects, leading to a general increase in accident and safety risk across consecutive nights^10,30^.

The aim of the current study was to characterise sleep-wake behaviour, circadian timing and alertness in shift workers in a healthcare setting. We specifically examined sleep and wake duration between shift transitions, temporal patterns of alertness and performance on the first and final night shifts (3^rd^, 4^th^ or 5^th^) in a sequence, and alertness and performance in relation to circadian timing during night shifts.

## Methods

### Participants

Fifty-two nurses and doctors (38 females, 41 nurses) aged between 22 and 64 (*n* = 50) were recruited from the Intensive Care Unit at Austin Health, Melbourne, Australia. Staff were recruited via e-mail advertising, scheduled in-service information sessions, flyer distribution within the unit and targeted recruitment on shift. No exclusion criteria were applied. Nursing staff engaged in generally irregular combinations of day (07:00–15:30 h), evening (13:00–21:30 h) and night shifts (21:00–07:30 h). Although nurses were typically required to work night shifts for a third of their annual roster, the distribution of shifts scheduled per fortnight varied. Some staff engaged in frequent rotations between day, evening and night shifts and others worked fixed night shifts for multiple consecutive weeks. During participation, the majority of nurses worked a sequence of day shifts and evening shifts followed by consecutive night shifts (n = 28). Additional participating nurses worked evening shifts followed by day shifts (n = 4), evening shifts prior to consecutive nights (n = 4), and 4 nurses worked permanent night shifts. One nurse was assessed on their day schedule after the night shifts. Doctors worked a fixed 4-week rotating roster consisting of 7 consecutive day shifts (08:00–21:00 h), 7 day offs and 7 consecutive night shifts (20:00–09:00 h), followed by 7 consecutive day offs.

Participants provided written informed consent prior to participation and received AU$100 upon completion of the study. The protocol was approved by Austin Health Human Research Ethics Committee and Monash University Human Research Ethics Committee. The study protocol was carried out in accordance with the standards set by the latest revision of the *Declaration of Helsinki*.

### Protocol

An online questionnaire was administered to assess demographic information, alcohol, caffeine and recreational drug consumption, sleep health, shift work history and medical history. Current medication and exercise during participation was recorded in weekly diaries.

Daily sleep-wake behaviour was assessed by wrist-worn actigraphs (Actiwatch Spectrum or Actiwatch Spectrum Plus, Respironics, Bend, OR, USA) and completion of daily sleep diaries. Diaries were completed daily to provide reports on sleep and wake timing and sleep duration, including any naps, in the 24 hours prior. Bedtime and wake time from sleep diaries were used to determine the daily analysis interval for objective actigraphic sleep parameters (Actiware 6 software, Respironics Inc, Bend, OR, USA). Actiware sensitivity was set to medium (40 activity counts) to determine an epoch as sleep or wake. Visual inspection of the graphical data (actogram) was conducted to identify discrepancies between reports of sleep timing in diaries and objective actigraphy, as has been conducted previously^31,32^. When the information from the sleep diary was not congruent with activity and light data from the actigraph, the analysis window was adjusted based on the following rules (7%, 26 sleep episodes). If a sustained substantial reduction in activity and light was ≥60 minutes prior to or after self-reported bedtime, the start of the analysis window was adjusted in 60-minute increments until it more closely aligned with the time of activity and light reduction. If a sustained substantial increase in activity and light was ≥60 minutes prior to or after self-reported wake time, the end of the analysis window was adjusted in 60-minute increments until it more closely aligned with the activity and light increase^31,32^. Light levels dropping to or rising above 10 lux was considered an indication of transition to/from sleep attempt. Data cleaning procedures were completed by two independent researchers with referral to a third researcher when conflicts were identified.

**Figure 1:**
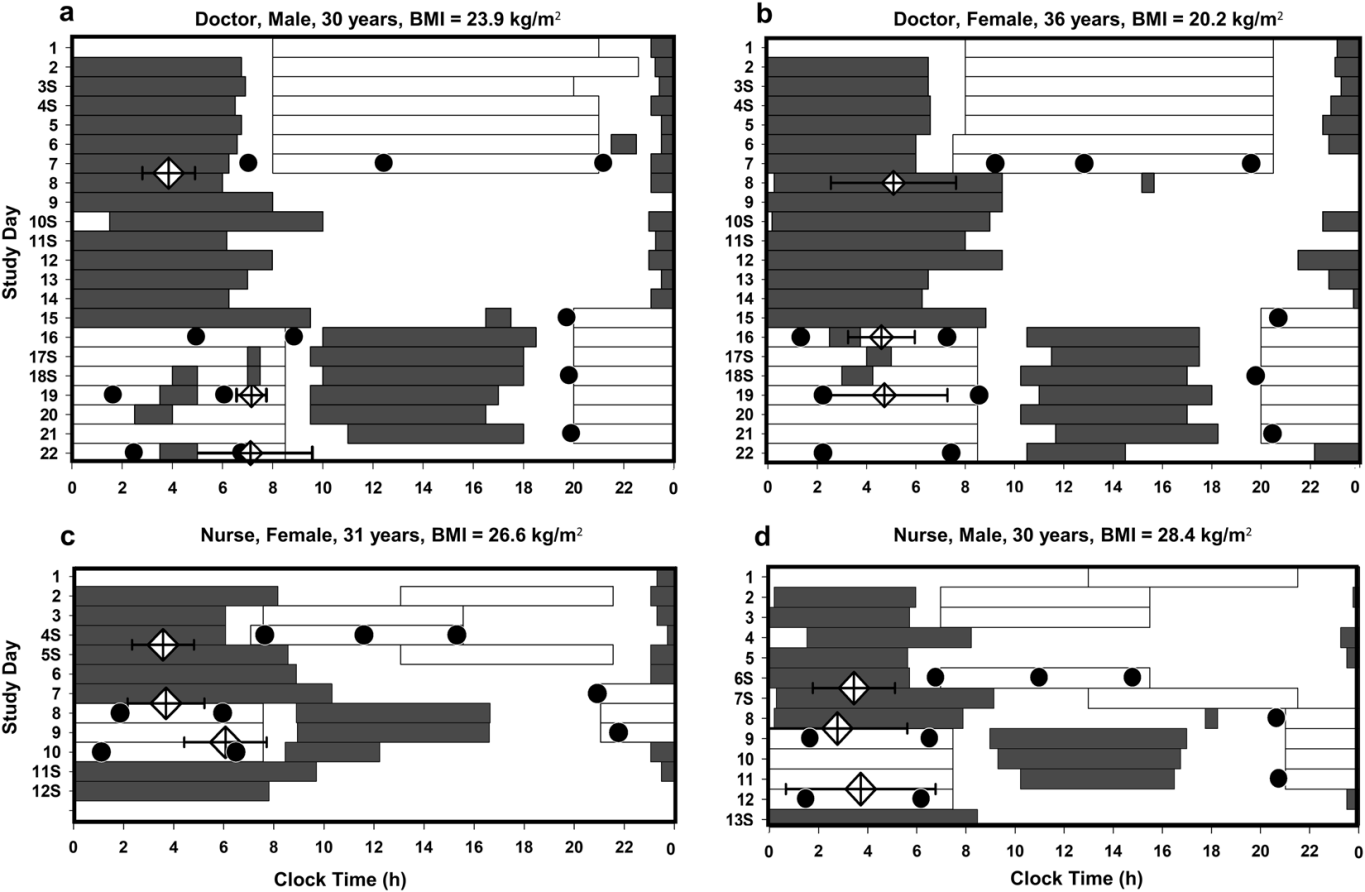
Raster plots of the timing of work (white bars), sleep (grey bars), in-shift alertness testing (closed circles) and aMT6s acrophase (diamonds) for two intensive care doctors (**a**,**b**) and nurses (**c**,**d**). Error bars on the diamonds represent 95% confidence intervals of the timing of acrophase. Doctors worked 7 day shifts, followed by 7 day offs and 7 consecutive night shifts. Nurses worked irregular rotations between day, evening and night shifts. Night shifts were sometimes associated with frequent napping during the shifts (**a**) and often a small delay in circadian timing (**a**,**c**). Sleep during the day was usually considerably shorter than night sleep (**b**,**c**). Sleep duration between evening and day shift was considerably truncated (**c**,**d**). Alertness and performance data from doctors tested on the 7^th^ night shift are not reported in this manuscript.

An alertness and performance test battery was administered on the last day shift of a diurnal shift sequence and during a sequence of night shifts. For nurses, the test battery was administered on the final day shift of the diurnal shift sequence, consisting of a combination of day shifts, evenings shifts and days off, followed by the first and the last (3^rd^, 4^th^ or 5^th^) consecutive night shift (Fig. 1). For doctors, the test battery was implemented on the last day shift of a 7-day sequence, and on the 1^st^, 4^th^ and 7^th^ consecutive night shift. Tests completed on the 7^th^ consecutive night shift for doctors have not been included in the current analyses due to the inadequate sample size (*n* = 7). For both doctors and nurses, tests were administered during the final day shift in a sequence to reduce any carry-over effects from prior night shifts. Tests were completed as close as operationally possible to the start, middle and end of each shift in a quiet office space within the ICU. A researcher was present for all testing to facilitate test completion and monitor compliance. The computerised test battery programmed in Matlab® (The Math Works Inc., USA) was implemented in the following order; Karolinska Sleepiness Scale (KSS), 5-minute visual Psychomotor Vigilance Test (PVT) and subjective ratings of concentration, difficulty and motivation.

The KSS measured subjective sleepiness on a scale of 1 = ‘very alert’ to 9 = ‘very sleepy, fighting sleep’^33^. The PVT was used as a measure of sustained attention. This task required participants to respond to a visual stimulus presented at random intervals (2–10 seconds), as quickly as possible by pressing on a response box key with their dominant thumb. The stimulus appeared as white digits, on a millisecond counter, on a black background^34^. During the 5-minute PVT, infrared reflectance (IR) oculography (Optalert™) was used to monitor eyes and eyelid movements^20,35^. The IR transducers fitted to spectacle frames are positioned towards the eyes to measure the relative velocity of blink durations and the closing and opening of the eyelid. A propriety algorithm using a combination of oculometric variables provides a minute by minute rating of drowsiness known as the Johns Drowsiness Scale (JDS)^36^. JDS scores range from 0–10, with higher JDS scores representing increased drowsiness.

A series of self-report questions based on a 9-point Likert scale, assessing level of concentration, task difficulty and motivation required to perform the PVT were administered at the end of each test session^37^.

Urine was collected to assess the rhythm of the urinary melatonin metabolite, 6-sulphatoxymelatonin (aMT6s), as a marker of circadian phase^38^. Participants collected urine samples for 24–48 hours, at approximately 4-hour intervals while awake and 8-hour intervals during sleep. Each participant completed a collection prior to and during the day shift and on the first and subsequent (3^rd^,4^th^ or 5^th^) night shifts on which the alertness and performance tests were administered. Participants recorded void times, collection times and volume of each urine sample. A 5 ml aliquot was stored at −20 °C and was subsequently assayed by radioimmunoassay for aMT6s (Adelaide Research Assay Facility, University of Adelaide) using reagents from Stockgrand Ltd (University of Surrey, Guildford, UK). The assay limit of detection was 0.5 ng/ml, intra-assay coefficient of variation (CV) was 7.4% for the first batch (*n = *376 samples) and 6.7% for the second batch (*n* = 881 samples). The inter-assay CVs were 8.7%, 6.3% and 7.7% at 2.7 ng/ml, 11.5 ng/ml and 19.7 ng/ml, respectively for the first batch and 14.9%, 3.5%, 5.4% at 5.7 ng/ml, 24.1 ng/ml and 41.9 ng/ml respectively for the second batch.

### Statistical analyses

Independent samples t-tests were performed to assess differences in the demographic variables and sleep health measures between doctors and nurses (see Table 1). Subjective (sleep diaries) and objective (actigraphic) estimates of total sleep time between shifts were calculated by summing the duration of all sleep episodes between the completion of one shift, and the start of the following shift. Total sleep time between consecutive days off was calculated from 12:00 h on one non-working day to 12:00 h on the following non-working day. Total sleep time between a day off and an evening or night shift was calculated from the 24 hours prior to the start of the evening or night shift. Total sleep time between consecutive day shifts, consecutive night shifts and consecutive days off was not different between doctors and nurses (Independent t-test, *p* > 0.05) and data were combined for analyses. Due to small sample sizes for the day to night shift (*n* = 5) and day to evening shift (*n* = 21) transitions, and lack of statistical difference in the amount of sleep obtained between these shift types (Independent t-test, *p* > 0.05), data were combined for analyses. Similarly, data were combined for the (1) evening to evening and evening to night shift transition and (2) off to evening and off to night shift transition. To control for the number of sleep contributions made by each participant, linear mixed model analysis with shift transition modelled as the fixed effect and participant as the random effect was performed to compare total sleep time between shift types. Pairwise comparisons using Bonferroni correction were conducted to compare sleep between consecutive day shifts and other shift types. A separate linear mixed model was implemented to compare differences in total sleep time prior to a 2^nd^, 3^rd^, 4^th^ and 5^th^ night shift. The night shift type [2^nd^, 3^rd^, 4^th^, 5^th^], was modelled as a fixed effect, with participant included as a random effect.

**Table 1:** Participant characteristics for intensive care doctors and nurses (n = 50) and summary of the consecutive night shifts worked by participants involved in the alertness and performance testing.

	Doctors (n = 11)	Nurses(n = 39)	Participants(*n* = 50)			
Demographics			n	Mean ± SD	Range	Doctors vs Nurses (*p*-value)
Sex (female)	5	31				**0.03**
Age (years ± SD)	32.7 ± 5.4	34.1 ± 10.6		33.8 ± 9.7	22–64	0.68
BMI (kg/m^2^ ± SD)	27.7 ± 8.1	24.6 ± 4.2		25.3 ± 5.4	16–44	0.09
Night shift experience (years ± SD))	7.1 ± 5.9	10.1 ± 7.7		9.5 ± 7.4	0–30	0.23
Caffeine consumption (Cups ± SD)) (Work Day)	1.1 ± 0.5	1.3 ± 0.7	50	1.3 ± 0.7	0–3	0.34
Caffeine consumption (Cups ± SD)) (Non-work Day)	0.9 ± 0.3	1.1 ± 0.6	50	1.1 ± 0.5	0–2	0.31
**Sleep-health measures**						
Insomnia Severity Index ± SD	6.3 ± 3.2	8.9 ± 5.6	50	8.3 ± 5.2	0–20	0.14
Epworth Sleepiness Scale ± SD	7.6 ± 4.6	6.4 ± 3.6	50	6.7 ± 3.8	0–15	0.36
<11	7	33	40			
≥11	4	6	10			
Morningness-Eveningness Questionnaire	32.4 ± 8.6	39.0 ± 6.4		37.5 ± 7.4	20–52	**0.01**
Definite evening type (16–30)	4	4	8			
Definite morning type (70–86)	0	0	0			
Pittsburgh Sleep Quality Index	6.0 ± 2.4	6.6 ± 2.8		6.5 ± 2.7	2–16	0.49
<5	4	6	10			
≥5	7	33	40			
**Alertness and performance testing** (Number of consecutive nights worked)						
3 night shifts	-	15				
4 night shifts	8	9				
5 night shifts	1	2				

The duration of wakefulness prior to work between different shift transitions was calculated as shift start time minus actigraphic sleep offset for the main sleep episode between shifts. Due to small sample sizes for the day to night (*n* = 5) and evening to night (*n* = 5) shift transitions and no significant difference in the wake duration between the transition types (Independent samples t-test; *t*(8) = 1.20, *p* = 0.270), these groups were combined for analyses. To assess time awake between shift transitions, linear mixed model analysis with the shift transition as a fixed effect and participant as a random effect was performed. Pairwise comparisons adjusted using Bonferroni correction were used to analyse differences between the combined day to night and evening to night shift types to other shift transition types.

To assess the impact of prior sleep and wake on test battery performance during shifts, participant sleep-wake characteristics in the 24 hours prior to the start of the day, first and subsequent (3^rd^, 4^th^ or 5^th^) night shift associated with testing were calculated from actigraphy. For this analysis only, missing actigraphic sleep data were substituted with data from sleep diaries as per previous studies^39^ to ensure sufficient power. Subjective sleep timing was determined from self-reported sleep onset and sleep offset times. Subjective total sleep time was calculated as the time between sleep onset and offset, minus the duration of any awakenings reported. Independent samples t-tests were used to assess differences in sleep prior to day shift and the first and subsequent night shift.

PVT reaction times (RT) less than 100 ms were excluded from the analyses^40^. The mean RT and fastest 10% of RTs were log transformed^40,41^ whilst lapses (defined as RT > 500 ms) were normalised using the equation √x + √(x + 1) as previously described^13^. Procedures for cleaning oculographic data are detailed in the Supplement. To assess temporal changes in KSS, PVT metrics, maximum JDS and subjective reports of motivation, task difficulty and concentration on the PVT, each shift (day, first and subsequent night shift) was split into equal thirds based on clock time (4.5 h bins for the day shift and 5 h bins for night shifts) such that data were allocated to one of three time bins for each shift (start, middle and end of shift) (Table 2). Additional details of data binning procedures and missing data imputation are provided in the Supplement. Overall, up to 11% of KSS data, 12% of PVT data, 14% of subjective ratings of performance data and 15% of oculography data were missing per shift.

**Table 2:** Doctors and nurses rostered shift times and ranges of times of the alertness and performance testing time during day and night shifts (n = 35).

Shift	Rostered Shift Times (h)		Test Time (Ranges; h)		
	Doctors	Nurses	Start	Middle	End
Day	08:00–21:00	07:00–15:30	05:00–09:29	09:30–13:59	14:00–18:30
Evening		13:00–21:30			
Night	20:00–09:00	21:00–07:30	18:30–23:29	23:30–04:29	04:30–09:30

Alertness and performance data collected from participants tested on a 3^rd^, 4^th^ or 5^th^ night shift were combined and analysed as a ‘final’ night shift to compare against the first night shift. Prior to combining, one-way ANOVAs confirmed that there were no differences in KSS or PVT mean reaction time between those tested on the 3^rd^, 4^th^ or 5^th^ night shift at the start, middle and end of shifts (*p* > 0.05). To examine the effect of shift type and time within shift on alertness and neurobehavioral performance, a 3 × 3 repeated measures ANOVA (time within shift [start, middle, end], shift type [day, first night, final night shift]) was used for KSS, PVT, maximum JDS and subjective reports of motivation, task difficulty and concentration. Where the sphericity assumption was not met, Greenhouse-Geisser’s correction was applied. Post-hoc comparisons using the Fisher’s Least Significant Difference method were performed where statistically significant effects of both shift and time within shift were found.

The timing of the peak (acrophase) of the aMT6s rhythm was determined using cosinor analysis as previously described^42^. To assess the impact of circadian phase on alertness and performance, KSS and PVT test times on the day, first and final night shift were presented as time since acrophase. Time since acrophase was defined as the difference (in decimal h) between the test time and an individual’s aMT6s acrophase on that shift, where positive values represent tests after acrophase and negative values represent tests before acrophase. Spearman’s rank order correlation was used to assess the relationship between time since acrophase and KSS, PVT mean reaction time and PVT lapses on each shift.

SPSS version 24.0 (SPSS Inc, Chicago, IL, USA) was used for imputing missing data and all data analyses. Unless otherwise stated, data are presented as mean ± SD values. Significance level was set at 0.05 for all statistical analyses.

## Results

### Participants

**Figure 2:**
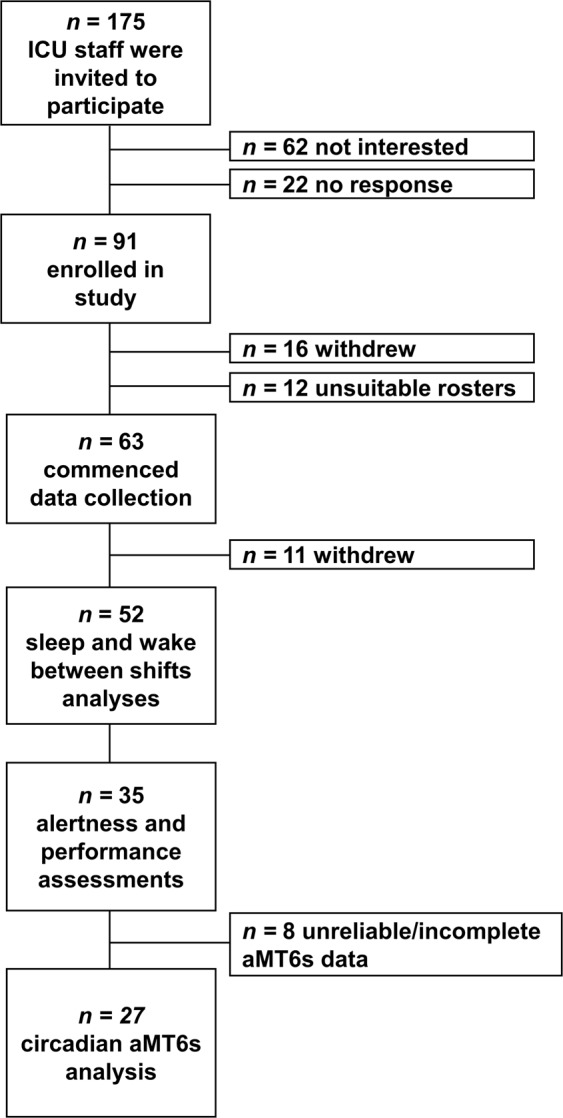
Recruitment Flowchart.

Of the 63 participants who commenced data collection (Fig. 2), 11 withdrew and 52 participants were included in the sleep between shifts and wake prior to work analyses. Two female nurses (1 working evening shifts followed by a sequence of day shifts, 1 working permanent night shifts) did not complete the demographics and sleep health questionnaire, hence demographic information is based on 50 participants (Table 1). Data from these 2 participants were included in the sleep between shifts and wake prior to work between shift types analyses. Due to technical difficulties with devices and participant non-compliance, actigraphic sleep data was not available for 8 participants. Of the 52 participants who contributed to the sleep and wake prior to work analyses, thirty-five (26 nurses, 9 doctors) contributed data to the analyses of alertness and performance on day compared to consecutive night shifts. One participant was tested on a day shift that occurred 5 days after the sequence of night shifts and one participant completed their first and final night shift testing on separate blocks of consecutive night shifts. Due to unexpected changes in rosters, one doctor was tested on the 5^th^ night shift instead of the 4^th^ night.

Due to the Optalert™ device not detecting eye and eyelid movements accurately following poor fitment of the glasses under the time constraints of the intensive care setting, data from 18 participants (16 nurses, 2 doctors) could not be included in the analysis. Due to corrected vision, an additional 8 participants (3 nurses, 5 doctors) were unable to use this device.

Of the 35 participants included in the alertness and performance analyses, 8 participants (23%) did not have adequate aMT6s data (incomplete collection or poor quality aMT6s rhythm determined by cosinor analysis) for the day, first and final night shift (Fig. 2). Of the remaining 27 participants, an additional 5 (14%) had inadequate aMT6s data for a day shift but first night shift data was used as a substitute. On the first night shift, an additional 10 (29%) participants had inadequate aMT6s data for the first night shift and day shift aMT6s data was used as a substitute. These substitutions were performed based on the assumption that acrophase timings would be the same immediately prior to the first night shift^42^. On the final night shift, 5 (20%) participants did not have adequate aMT6s data, and data were excluded.

### Demographics

Fifty-two participants (41 nurses, 11 doctors) completed data collection. Participants (*n* = 50) were aged 22–64 years (33.8 ± 9.7 years, mean ± SD), with a body mass index (BMI) ranging from 15.9–43.6 kg/m^2^ and had an average of 9.5 ± 7.4 years of night work experience (Table 1).

### Sleep between shift types

Linear mixed models revealed a significant difference in total sleep time between shift types for sleep measured using sleep diaries (*F*(6, 479) = 36.42, *p* < 0.001) and actigraphy (*F*(6, 385) = 37.25, *p* < 0.001). Pairwise comparisons with Bonferroni correction indicated no significant difference in total sleep time between consecutive day shifts (actigraphy: 5.83 ± 0.92 h; sleep diary: 6.71 ± 0.96 h) and consecutive night shifts (actigraphy: 5.74 ± 1.30 h; sleep diary: 6.18 ± 1.52 h) (Table 3). Compared to consecutive day shifts (6.71 ± 0.96 h), least sleep was obtained between an evening and a day shift the following morning based on subjective sleep (5.66 ± 0.92 h, *p* = 0.005). Although sleep measured by actigraphy was shorter between an evening and day shift (5.20 ± 0.90 h) compared to consecutive day shifts (5.83 ± 0.92 h), this difference was not statistically significant (*p* > 0.050, Table 3). On work days, sleep duration was longest between day to night and day to evening shift transitions (actigraphy: 7.96 ± 1.40 h; sleep diary: 8.09 ± 2.19 h). Linear mixed model analysis demonstrated no differences in total sleep time prior to a 2^nd^, 3^rd^, 4^th^ or 5^th^ night shift based on both objective total sleep time (*F*(3, 81) = 0.02, *p* = 0.990) and subjective total sleep time (*F*(3, 122) = 0.84, *p* = 0.480).

**Table 3:** Total sleep time between different shift transitions and between consecutive night shifts calculated using wrist-worn actigraphs (n = 44) and sleep diaries (n = 52).

	Actigraphic Sleep(n = 44)			Subjective Sleep(n = 52)		
Shift Pattern	Total sleep time between shifts (Mean ± SD) (h)	Range (h)	Sleep entries (n)	Total sleep time between shifts (Mean ± SD) (h)	Range (h)	Sleep entries (n)
Day to Day	5.83 ± 0.92	3.15–7.48	57	6.71 ± 0.96	4.00–8.62	68
Night to Night	5.74 ± 1.30	2.30–8.57	107	6.18 ± 1.52	1.83–10.33	135
Day to Night/Day to Evening	7.96 ± 1.40**	5.22–11.08	26	8.09 ± 2.19**	2.50–11.98	31
Evening to Day	**5.20 ± 0.90**	3.15–7.28	33	5.66 ± 0.92**	4.00–7.42	38
Evening to Night/Evening to Evening	7.53 ± 1.60**	5.43–10.77	18	7.92 ± 1.35*	5.87–11.08	22
Off to Off	7.40 ± 1.28**	3.75–10.17	99	8.27 ± 1.72**	1.83–14.42	128
Off to Night/ Off to Evening	8.00 ± 1.98**	2.42–12.22	45	8.50 ± 1.86**	3.77–12.40	57
1^st^ to 2^nd^ Night	5.76 ± 1.24	2.30–7.80	37	5.94 ± 1.61	1.83–8.13	47
2^nd^ to 3^rd^ Night	5.69 ± 1.28	2.83–7.48	36	6.10 ± 1.52	2.17–8.88	46
3^rd^ to 4^th^ Night	5.70 ± 1.23	3.92–8.57	18	6.55 ± 1.49	4.17–10.33	23
4^th^ to 5^th^ Night	5.69 ± 1.89	2.77–8.10	9	6.35 ± 1.77	3.63–9.72	10

*p < 0.05 and **p ≤ 0.005 indicate significant differences in sleep duration compared to day to day transition after adjusting for Bonferroni correction.

### Duration of wake prior to work between shift types

Linear mixed model analysis revealed a significant difference in the duration of wake prior to work between shift types (*F*(7, 286) = 124.05, *p* < 0.001). Pairwise comparisons with Bonferroni correction revealed that the duration of wake was longest for night shifts preceded by a day or evening shift (12.10 ± 1.99 h; Table 4). Day shifts preceded by evening shifts (1.68 ± 0.52 h) or day shifts preceded by day shifts (1.78 ± 0.78 h) were associated with the shortest wake durations.

**Table 4:** Duration of wake prior to work between shift types calculated using wrist-worn actigraphs (n = 44).

Shift Pattern	Time awake from main sleep (Mean ± SD) (h)	Range (h)	Sleep entries(n)
Day to Night/Evening to Night	12.10 ± 1.99	9.08–15.12	10
Night to Night	4.70 ± 2.04*	0.45–9.03	107
Evening to Day	1.68 ± 0.52*	0.52–3.12	33
Day to Day	1.78 ± 0.78*	0.52–5.62	57
Day to Evening	4.78 ± 1.61*	2.05–8.20	21
Evening to Evening	5.26 ± 1.63*	3.02–8.00	13
Off to Evening	4.83 ± 1.56*	2.20–8.27	17
Off to Night	12.15 ± 3.38	2.02–17.18	28

*p < 0.001 indicate significant differences in wake duration compared to the day to night/evening to night shift transitions after adjusting for Bonferroni correction.

### Sleep-wake information prior to alertness and performance testing

On average, compared to day shift (05:32 ± 00:40 h), participants woke significantly later prior to the first (08:23 ± 01:41 h, *t*(60) = −8.91, *p* < 0.001) and final night shift (16:06 ± 01:32 h, *t*(63) = −36.07, *p* < 0.001; Table 5). Total sleep time was longer in the 24 h prior to the first (8.19 ± 1.41 h, *t*(60) = −7.47, *p* < 0.001) and final night shift (6.36 ± 1.18 h, *t*(63) = −2.12, *p* = 0.040) compared to the day shift (5.74 ± 1.17 h). More napping occurred prior to the first (*t*(60) = −3.14, *p* = 0.003) and final night shift (*t*(63) = −3.58, *p* = 0.001) compared to the day shift. Participants woke earlier prior to the first night shift (08:23 ± 01:41 h) compared to the final night shift (16:06 ± 01:32 h, *t*(59) = −18.73, *p* = 0.001). The duration of wake since main sleep was longer prior to the start of the first night (12.74 ± 2.02 h) compared to the final night shift (5.25 ± 2.12 h, *t*(52) = 13.26, *p* < 0.001, Table 5). Total sleep time in the 24 hours prior to the first night shift (8.19 ± 1.41 h) was longer compared to the final night shift (6.36 ± 1.18 h, *t*(59) = 5.50, *p* < 0.001). No differences in nap duration were observed between the first and final night shift (*t*(59) = −0.09, *p* = 0.930).

**Table 5:** Participant sleep-wake characteristics in the 24 hours prior to a day, first and final (3^rd^/4^th^/5^th^) night shift calculated from actigraphy with sleep episodes identified by sleep diary entries. Missing actigraphic sleep-wake information has been substituted with data from sleep diaries. There were no sleeps with a duration <15 minutes.

	Mean ± SD		Mean ± SD		Mean ± SD		First vs Final night shift
	Day shift	n	First night shift	n	Final night shift	n	*p*
Wake time (hh:mm)	5:32 ± 00:40	33	8:23 ± 01:41**	29	16:06 ± 01:32**	32	**0.001**
TST, all sleeps (h)	5.74 ± 1.17	33	8.19 ± 1.41**	29	6.36 ± 1.18*	32	**0.001**
Nap (sleep <120 minutes)	0	33	19.79 ± 36.23**	29	20.56 ± 33.05**	32	0.931
Time awake from main sleep^#^ (sleep ≥120 minutes) (h)	2.53 ± 1.31	33	12.74 ± 2.02**	26	5.25 ± 2.12**	28	**0.001**
Time awake from last sleep^#^ (sleep ≥15 minutes) (h)	2.53 ± 1.31	33	9.76 ± 4.14**	26	4.77 ± 1.86**	28	**0.001**

*p < 0.05 and **p < 0.005 indicate significant differences from day shift.

^#^Indicates time elapsed since main sleep or last sleep prior to the start of a shift.

**Figure 3:**
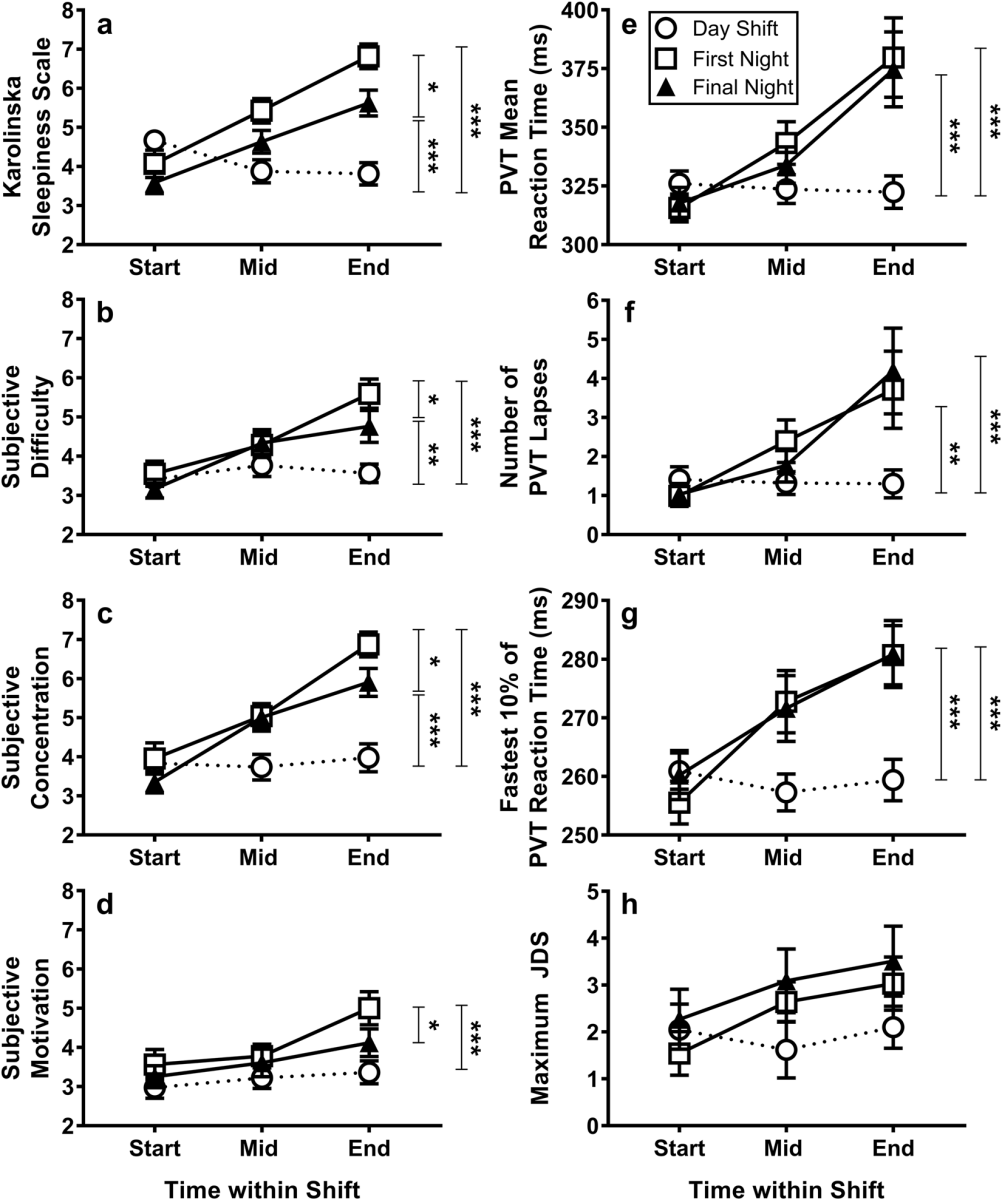
Alertness, performance and subjective report for PVT performance of intensive care workers on a day shift and first and final (3^rd^/4^th^/5^th^) night shift as measured by (**a**) Karolinska Sleepiness Scale, (**b**) subjective difficulty, (**c**) subjective concentration, (**d**) subjective motivation, (**e**) PVT mean reaction time (**f**) number of PVT lapses (**g**) PVT fastest 10% of reaction times and (**h**) maximum JDS (*n* = 9) during the 5-minute PVT. In all figures, higher values represent poorer outcomes. Higher values indicate increased impairment on all measures. Astericks indicate differences between shifts only at the end of shifts *p ≤ 0.05, **p ≤ 0.005, and ***p ≤ 0.001 indicates the differences in alertness and performance at the end of shifts between day and first night, day and final night and between first and final night shift.

*PVT Performance*. Repeated measures ANOVA demonstrated significant main effects of shift (*F*(2, 62) = 7.23, *p* = 0.002), time within shift (*F*(1.2, 38.4) = 26.38, *p* = 0.001), and a shift by time interaction (*F*(2.7, 83.2) = 16.04, *p* = 0.001) for PVT mean RT. On both the first and final night shift, a consistent increase in mean RT was observed from the start to middle (*p* < 0.001) and middle to end (*p* ≤ 0.001) of shift (Fig. 3e). Post-hoc comparisons using Fisher’s Least Significant Difference revealed that mean RT at the end of shift was longer on the first (*p* < 0.001) and final night shift (*p* < 0.001) compared to the day shift. Between the first and final night shift, there were no significant differences in mean RT at the start, middle or end of shift (Fig. 3e). This was consistent for the fastest 10% of RTs and number of lapses (Fig. 3f,g).

### Subjective sleepiness

Repeated measures ANOVA revealed a main effect of shift (*F*(2, 64) = 14.17, *p* < 0.001), time within shift (*F*(2, 64) = 29.09, *p* < 0.001), and a shift by time interaction (*F*(4, 128) = 14.09, *p* < 0.001) for KSS ratings (Fig. 3a). On both the first and final night shift, KSS ratings were higher at the middle and end of shift compared to the start (all *p* < 0.010). Pairwise comparisons with Fisher’s Least Significant Difference, revealed that during the middle (*p* = 0.040) and end of shift tests (*p* = 0.008), first night KSS ratings were consistently higher than on the final night shift. Compared to the final night shift (3.59 ± 1.60), day shift KSS was higher at the start of shifts (4.67 ± 1.36, *p* = 0.005). There were no differences in start shift KSS ratings between the day and first night shift (*p* = 0.170). Post hoc tests were conducted to examine whether KSS at the start of the day shift was influenced by working an evening shift the night before. Independent samples t-tests demonstrated that there were no differences in KSS ratings between those who worked a day (4.80 ± 1.81) or an evening shift prior (4.59 ± 1.19, *t*(22) = 0.34, *p* = 0.730) to the day shift. The sleep onset time on the night prior to the tested day shift did not differ between those who worked an evening shift (23:22 ± 00:34 h) or a day shift prior (23:25 ± 01:10 h, *t*(28) = −0.17, *p* = 0.86).

### Subjective difficulty, concentration and motivation

Subjective ratings of difficulty on the PVT revealed an effect of shift (*F*(1.7, 52.4) = 6.55, *p* = 0.005), time within shift (*F*(1.6, 50.8) = 22.17, *p* < 0.001), and a shift by time interaction (*F*(4, 124) = 6.35, *p* < 0.001). At the end of shift, difficulty ratings were higher on the first night shift compared to the final night shift (*p* = 0.03) (Fig. 3b).

Repeated measures ANOVA revealed a significant effect of shift (*F*(2, 62) = 15.69, *p* < 0.001), time within shift (*F*(1.6, 48.1) = 42.55, *p* < 0.001), and a shift by time interaction (*F*(4, 124) = 7.99, *p* < 0.001) on subjective ratings of concentration on the PVT (Fig. 3c). Concentration ratings in the middle of the first (*p* = 0.005) and final night shift (*p* = 0.007) were higher compared to the day shift. At the end of shift, first night concentration ratings were higher than the final night shift (*p* = 0.020).

ANOVA demonstrated a significant effect of shift (*F*(2, 62) = 7.76, *p* = 0.001) and time within shift (*F*(2, 62) = 10.73, *p* < 0.001) on subjective motivation (Fig. 3d). There was no significant shift by time interaction observed (*F*(3.2, 97.9) = 2.11, *p* = 0.101). On both the first (*p = *0.004) and final (*p* = 0.013) night shift, post-hoc comparisons confirmed that motivation ratings at the end of shift were higher compared to the start of shift. At the end of shift, pairwise comparisons (Fisher’s Least Significant Difference) confirmed that motivation ratings were higher on the first night shift compared to the final night shift (*p* = 0.020).

### Johns Drowsiness Scale

The maximum JDS scores from the Optalert ™ device worn during the 5-minute PVT demonstrated a main effect of time (*F*(2, 16) = 4.96, *p* = 0.020) with higher values at the middle and end of shifts (Fig. 3h). There was no main effect of shift or a shift by time interaction effect for maximum JDS scores.

### Circadian phase

**Figure 4:**
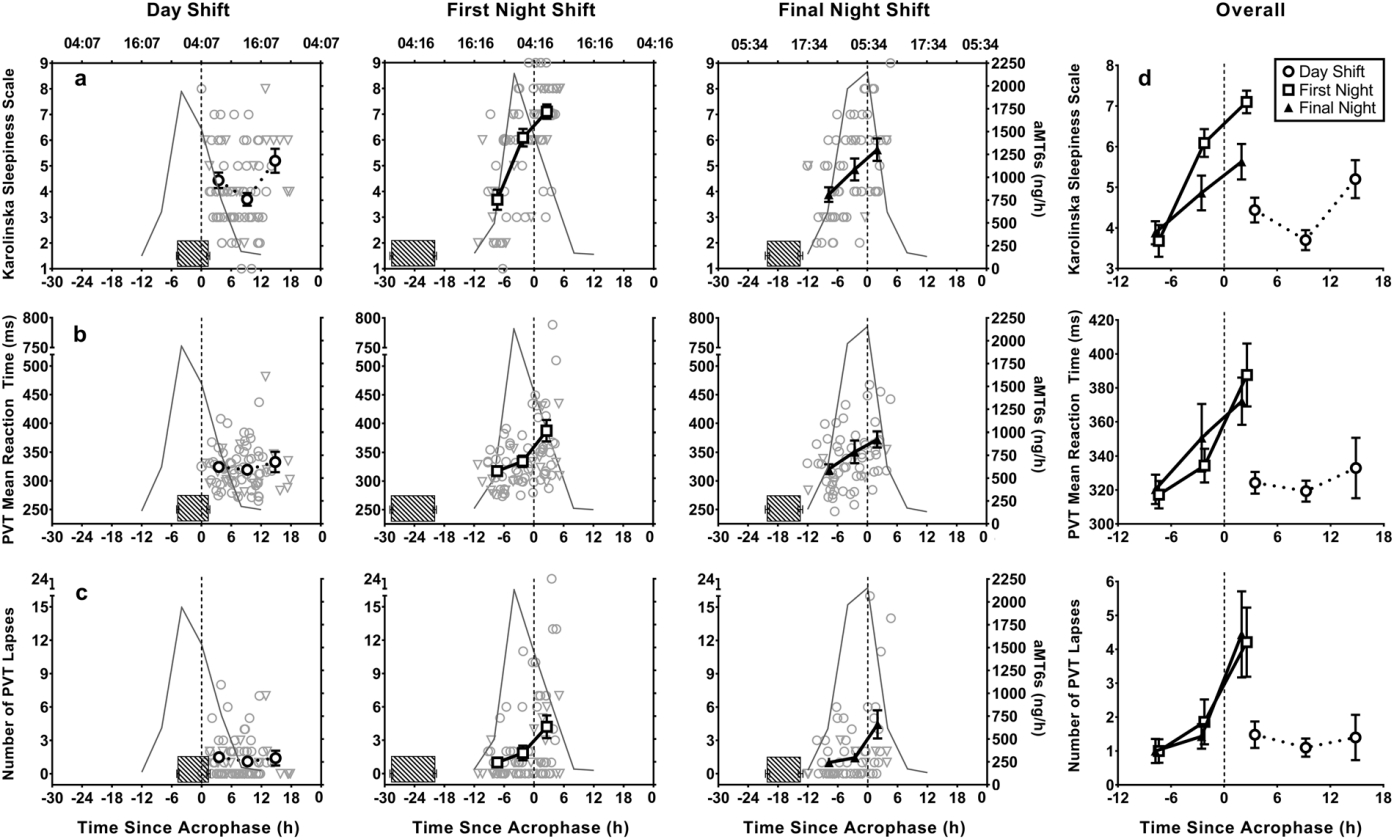
Alertness and performance of intensive care workers relative to aMT6s acrophase on a day (left), first (middle) and final (3^rd^–5^th^) night shift (right) shown by the (**a**) Karolinska Sleepiness Scale, (**b**) PVT mean reaction time, (**c**) number of PVT lapses. Zero on the x-axis indicates time of acrophase, positive values represent tests before acrophase (day shift: 0–6 h, 6–12 h, 12–18 h; night shift: −12 to −5 h, −5–0 h, 0–5 h) Grey curves represent aMT6s rhythms averaged for all individuals. Striped horizontal bars represent the average main sleep prior to the start of a shift. Right panel (**d**) presents combined graphs of the changes in KSS, PVT mean reaction time and PVT lapses across the day, first and final night shift.

Over the course of a day shift, no significant correlations were found between time since acrophase and PVT mean RT andPVT lapses. On the first (RT: *r*_*s*_ = 0.38, *p* = 0.001; Lapses: *r*_*s*_ = 0.300, *p* = 0.010) and the final night shift (RT: *r*_*s*_ = 0.36, *p* = 0.005; Lapses: *r*_*s*_ = 0.39, *p* = 0.002), a medium positive relationship^43^ between time since acrophase and PVT mean RT and PVT lapses was observed, such that performance was poorer when tests were administered closer to the time of aMT6s acrophase (Fig. 4). Similarly, subjective sleepiness showed no significant correlation between KSS scores and the time since acrophase for the day shift (*p* = 0.830) but did show a strong positive relationship with time since acrophase on the first (*r*_*s*_ = 0.63, *p* < 0.001) and final night shift (*r*_*s*_ = 0.490, *p* < 0.001) such that subjective sleepiness was more impaired closer to the timing of aMT6s acrophase (Fig. 4).

## Discussion

This study demonstrates that alternating between different shift types has a significant effect on the duration of sleep and wake between shifts in intensive care workers. Sleep was most restricted between consecutive night shifts, consecutive day shifts and particularly between evening and day shifts. The first night shift in a sequence was associated with an extended period of wake before the shift. Alertness and performance were most impaired on nights compared to day shifts, with most impairment observed at the end of night shifts when tests were administered at an adverse circadian phase, closer to the timing of aMT6s acrophase. Despite a similar degree of performance impairment on the objective measures for the first and final night shifts, subjective ratings of sleepiness, task difficulty, concentration and motivation indicated increased impairment on the first night shift only. These findings support the concept that rotating shift work significantly alters sleep-wake behaviour between different shift types, and that both sleep and circadian factors drive alertness impairment on night shifts.

Sleep duration was particularly restricted between evening and day shifts (‘quick returns’). A common pattern of shifts in nursing, the late end time of the evening shift and the early start time of the subsequent day shift restrict the total break duration, to only 9.5 hours. The current data indicates that these quick returns should be avoided as they provide limited opportunity for recovery sleep. Of the different types of shift transitions worked by intensive care workers, it was expected that the most sleep would be obtained between consecutive day shifts as sleep occurs during the biological night where the circadian drive promotes sleep^44^. Sleep between consecutive day shifts, however, was not different to sleep between consecutive night shifts, in line with the findings of another study of healthcare workers where nurses also had an early start (07:00 h) to day shifts^6^. While these findings suggest that the reduced total sleep time between day shifts is likely to be the result of an early shift start, it also reflects on the competing pressures on healthcare workers’ sleep time during ‘regular’ shifts as well as night shifts. Compared to the break duration in the evening to day shift transition (9.5 h), the break duration between consecutive day shifts (nurses: 15.5 h; doctors: 11 h) was longer. While longer break durations generally provide greater opportunity for sleep, there was only 40–60 minutes of difference in sleep duration between evening-to-day shift transitions and consecutive day shifts (subjective sleep: 5.7 vs 6.7 h; objective sleep: 5.2 vs 5.8 h). The short sleep between consecutive day shifts could have resulted from reduced sleep opportunity due to social influences or domestic commitments, or perhaps the result of an expectation of being sleepier on nights, such that there is less importance given to prioritizing sleep on day shifts. On multiple occasions (43% of all main sleeps, sleep ≥120 minutes), total sleep time was below the recommended 7 h for optimal functioning^45^, supporting findings from other studies that healthcare workers are frequently exposed to chronic partial sleep deprivation^7,46^. In the current population of intensive care workers however, insufficient recovery sleep on work days appeared to be compensated to some extent with longer recovery sleep (>7 h) during non-work days, and on days when shifts started later in the day (e.g. evening shifts).

Despite short sleep prior to day shift tests, alertness and performance were more impaired on night shifts compared to day shifts. On both the subjective and objective measures, increased impairment was observed at the middle and end of both the first and final night shift compared to the start. Given the timing of tests in relation to aMT6s acrophase, it was likely that KSS ratings and PVT performance were consistently better at the start of night shifts due to the effect of the wake maintenance zone (WMZ), a 2–3 h window of reduced sleep propensity which occurs several hours before habitual sleep onset^47^. As expected, circadian phase had a significant effect on alertness and performance on both the first and final night shift, demonstrating that tests administered at an adverse circadian phase, defined as closer to the timing of aMT6s acrophase, were associated with poorer alertness and performance outcomes^36,48–50^. As demonstrated previously, minimal adaptation of circadian phase to night shift was observed, resulting in work schedules that coincided with the maximal circadian drive for sleepiness^42^. The combined multiplicative effects of being required to perform at an adverse circadian phase when coupled with the increased time awake associated with the latter half of a night shift, makes this time particularly vulnerable to sleepiness-related performance impairments and increases the risks associated with accidents and injuries^30,51^.

In shift work settings, we would expect increased usage of naps and caffeine to alleviate the high levels of sleepiness experienced during night work^52,53^. In this population of healthcare workers, napping on nights was minimal, and on work-days, caffeine consumption was low. Significant impairment on night shifts was evident on all measures except JDS scores measured via oculography. Despite the smaller sample size which could have limited the significance of the results, drowsiness tended to be higher towards the middle and end of shifts and a trend for more impairment on nights was also evident^36^.

In parallel with findings from other shift work studies, temporal patterns of alertness and performance across a day shift remained relatively stable^6,19^. Subjective sleepiness (KSS) was most impaired at the start of day shifts compared to the middle and end of shifts. We expected participants who worked an evening prior to the day shift to have contributed significantly to the higher KSS ratings at the start of day shifts as their opportunity for sleep was shorter, but this was not apparent. Furthermore, between those who worked a day or evening shift prior to the day shift, no differences in sleep onset time was observed on the night sleep prior. Thus, higher KSS ratings at the start of day shifts were likely due to the combined effects of an early shift start, short sleep prior, circadian phase and sleep inertia^54^.

The first night shift in a sequence is often preceded by prolonged wakefulness^15^. By the end of the first night, the continuous time awake often reaches 24 hours or more, and coincides with the circadian nadir for alertness, a time associated with an increased risk of accidents and errors^51,55^. On subsequent night shifts, the duration of wake prior to the shift is shorter but the effects of sleep restriction are cumulative (causing sleepiness and performance impairments over successive days of reduced sleep duration)^56,57^. In the current study, the first night was preceded by 12.7 h of wakefulness on average, which was more than twice the duration spent in wakefulness prior to the start of the final night shift (5.3 h). Combined with the effect of working during the circadian low, and increasing homeostatic sleep pressure, we would expect to see differences in alertness and performance between the first and the final night shift. Contrary to studies which have demonstrated increased impairment on the first night on both subjective and objective measures of alertness and performance^16,19^, we only observed significant first night impairment on the subjective measures, specifically at the end of the first night shift. While this reflects on the high level of anticipation associated with working on several consecutive night shifts, likely related to a perceived state of wellbeing^58^, it also suggests that subjective measures are likely to overestimate actual performance impairments^58^. An alternative interpretation is that, given the well-established and increasing mismatch between subjective sleepiness and objective performance over several days of chronic sleep loss^59,60^, the participants rated their subjective sleepiness accurately with respect to objective performance on the first night due to their acute sleepiness but were unable to do so over subsequent nights.

While this study was conducted in the field and has high ecological validity, applicability of these findings to settings outside of the healthcare environment or to operations implementing alternative rosters (i.e. fixed rather than rotating schedules) may be limited. In this study, we did not consider the influence of a second job, which may have been facilitated by long periods of time-offs and a large number of days off ^61^. We also note that we did not control for menstrual phase in this female dominated population, although it has shown to impact on attentional failures when accompanied by acute sleep deprivation^61^. Due to the variability in shift patterns in this setting, data from participants working 3, 4 and 5 night shifts were combined to assess alertness and performance after several consecutive night shifts compared to a first night shift. Although preliminary analyses revealed data were similar between participants tested on the 3^rd^, 4^th^ and 5^th^ consecutive night shifts, future research should assess alertness and performance on each of these nights separately to examine the impact of each additional night shift. Due to limited data from doctors working 7 consecutive night shifts, the impact of working 7 nights on alertness and performance could not be examined. Future research should examine the impact of working beyond 3 to 5 night shifts to assess the alertness and performance consequences in populations engaging in longer blocks of nights. It was the primary aim of this study to compare alertness and performance on day shifts to night shifts and it was not within the scope to assess waking function on evening shifts. Although the current protocol was able to assess sleep-wake behaviour associated with evening shifts, further assessment of the temporal changes in alertness and performance on evening schedules is warranted.

The current study extends previous simulation^16,36,41^ and operational studies^62,63^ by comparing different shift types and transitions, the impact of multiple night shifts and examining temporal changes in alertness and performance during these shifts. Our data suggests that early day shift start times, and evening-to-day shift transitions (‘quick returns’) should be avoided within safety-sensitive environments. Consideration of shift rotation patterns, shift duration and duration of time off between shifts should all be paramount when designing shift work rotas. The current study demonstrates the need for alertness countermeasures during night shift work. In addition to, but not as a substitute for improved shift patterns, mandatory breaks, and the strategic use of caffeine and naps could help alleviate sleepiness and fatigue during work shifts^53,64^. Future research should assess operationally relevant errors during the course of the night shift to understand more directly relevant performance impairment during shifts^26,51,65,66^. To be able to implement targeted interventions to help enhance alertness and performance during shifts, significant focus should be placed on the development and implementation of tools to assess fitness-for-duty during work shifts. This study has identified higher risk schedule patterns that limit sleep and has provided an understanding of alertness and performance impairment during consecutive night work, providing operational evidence for optimising shift designs.

## Supplementary information

Supplementary Material

## Data Availability

Materials and data in this publication can be requested via the CRC for Alertness, Safety and Productivity (Alertness CRC) by emailing inquiries@alertnesscrc.com.
