# Supplementary material for: The Impact of Shift Work on Sleep, Alertness and Performance in Healthcare Workers
Source: Sci Rep. 2019 Mar 15;9:4635. doi: 10.1038/s41598-019-40914-x (PMC6420632; doi:10.1038/s41598-019-40914-x)
Supplement: Supplementary file 1 — Supplementary Material [file 41598_2019_40914_MOESM1_ESM.doc]

**The Impact of Shift Work on Sleep, Alertness and Performance in Healthcare Workers.**

Saranea Ganesan^1,2^, Michelle Magee^1,2^, Julia E. Stone^1,2^, Megan D. Mulhall^1,2^, Allison Collins^3^, Mark E. Howard^1,2,3^, Steven W. Lockley^1,2,4,5^, Shantha M.W Rajaratnam^1,2,4,5^, *Tracey L. Sletten^1,2^

^1^Cooperative Research Centre for Alertness, Safety and Productivity, Melbourne, Victoria, Australia

^2^Monash Institute of Cognitive and Clinical Neurosciences and School of Psychological Sciences, Monash University, Clayton, Victoria, Australia

^3^Institute for Breathing and Sleep, Austin Health, Heidelberg, Victoria, Australia

^4^Division of Sleep and Circadian Disorders, Department of Medicine, Brigham and Women’s Hospital, Boston, MA, USA

^5^Division of Sleep Medicine, Harvard Medical School, Boston, MA, USA

*Corresponding author: Tracey L. Sletten, Monash Institute of Cognitive and Clinical Neurosciences and School of Psychological Sciences, Monash University, 270 Ferntree Gully Road, Notting Hill, VIC, 3168, Australia. [Email: tracey.sletten@monash.edu]

**Supplementary methods:**

**Oculography data cleaning procedure.**

Oculography data were visually inspected in 1-min epochs to ensure signal quality. Poor quality, defined as more than 50% of information within a 1-min epoch displaying an unclear indication of eye and eye lid movements based on the frequency, amplitude and velocity of the signal were excluded^1^. To ensure inter-rater reliability, 25% of all the oculographic data was re-inspected by a second researcher (*r* = .87). Maximum JDS during the 5-minute PVT was computed^2^.

**Additional data cleaning procedures and missing data imputation methods for the alertness and performance test battery data.**

To account for some tests being completed before or after the scheduled shift times, tests were binned based on the clock time they occurred. Tests which occurred outside of the time ranges specified in Table 2 were not included in the analyses. If two tests from one participant occurred within the same time bin, the test which occurred closer to the average test time for that bin was used (day shift: mid-shift time bin (*n* = 2), end shift time bin (*n* = 1); first night shift: mid-shift time bin (*n* = 1), end shift time bin (*n* = 3); last night shift: mid-shift time bin (*n* = 1). The test in the adjacent time bin was then considered missing. If a participant did not complete a test in the specified time bin, data were imputed using the expectation maximisation method^3^. When two out of the three tests (per shift type) were missing for a participant, data was excluded (< 15% of data was missing within each shift type for each measure). Missing data from the day, first and final night shift was deemed random based on Little’s MCAR tests performed on the KSS, PVT, maximum JDS and subjective reports of motivation, task difficulty and concentration^4^.

1 Anderson, C., Chang, A.-M., Sullivan, J. P., Ronda, J. M. & Czeisler, C. A. Assessment of drowsiness based on ocular parameters detected by infrared reflectance oculography. *Journal of Clinical Sleep Medicine* **9**, 907 (2013).

2 Ftouni, S. *et al.* Objective and subjective measures of sleepiness, and their associations with on-road driving events in shift workers. *Journal of Sleep Research* **22**, 58-69 (2013).

3 Hair, J. F. J., Black, W. C., Babin, B. J. & Anderson, R. E. *Multivariate Data Analysis*. 7th edn, (Pearson Education Limited, 2013).

4 Little, R. J. A test of missing completely at random for multivariate data with missing values. *Journal of the American Statistical Association* **83**, 1198-1202 (1988).
